# Leptospirosis and coinfections leading to fatal multiple organ and system failure

**DOI:** 10.1590/S1678-9946202567026

**Published:** 2025-04-14

**Authors:** Luiza Schettini Marotto, Marcia Schettini Marotto, Tomás Zecchini Barrese, Cinthya dos Santos Cirqueira Borges, Juliana Mariotti Guerra, Leonardo José Tadeu de Araújo, Camila Santos da Silva Ferreira, Amir Nassar, Paulo Cesar Fumagalli Marotto

**Affiliations:** 1 Hospital Municipal Antonio Giglio, Osasco, São Paulo, Brazil; 2 Instituto Adolfo Lutz, Departamento de Patologia, São Paulo, São Paulo, Brazil; 3 Instituto de Assistência Médica ao Servidor Público Estadual de São Paulo, Programa de Pós-Graduação em Ciências da Saúde, São Paulo, São Paulo, Brazil; 4 Universidade Nove de Julho (Campus Vergueiro), Faculdade de Medicina, São Paulo, São Paulo, Brazil

**Keywords:** Leptospirosis, COVID-19, Human rhinovirus, Dengue, Coinfection, Multiple organ failure

## Abstract

Coinfection with leptospirosis and other infectious agents pose major challenges in medical practice, often due to difficulties in isolating these agents, symptoms overlap, and lack of specific investigation protocols in areas with emerging and re-emerging diseases. Consequently, knowledge regarding these coinfections and their impact on clinical outcomes are limited. A previously healthy 33-year-old woman, with no history of chronic or malignance diseases, was admitted with febrile icteric illness, pulmonary hemorrhage, acute kidney injury, thrombocytopenia, and shock. Leptospirosis, COVID-19, human rhinovirus, and dengue in the acute phase were clinically and pathologically diagnosed. Multiple coinfections can rapidly lead to multiple organ and system failure, often resulting in a fatal outcome.

## INTRODUCTION

Leptospirosis is a disease caused by a spirochete (*Leptospira* spp.), primarily transmitted via urine of infected mammals (e.g., pets, farm animals, wild mammals).

While many wild and domestic animals can serve as reservoirs, the brown rat (*Rattus norvegicus*) is the most significant source of human infections^1,2^. Indirect transmission, such as contact between the host's skin and contaminated soil, is also common. The disease is endemic in Brazil, especially during periods of rain and flooding in urban areas. The fatality rate for Weil's disease and pulmonary hemorrhage syndrome exceeds 10% and 70%, respectively^1,2^.

COVID-19 is a viral disease caused by SARS-CoV-2, primarily transmitted via aerosols that enter the respiratory tract (i.e., the oropharynx)^3^. The fatality rate is 1.4%, though it is higher in individuals older than 50 years^3^. While its mortality rate is lower compared to previous coronavirus outbreaks, such as the 2002 SARS-CoV and 2012 MERS-CoV outbreaks, SARS-CoV-2 has higher transmissibility^3^. Although incidence has decreased since the official end of the pandemic and the rollout of booster vaccines to improve coverage, COVID-19 cases persist globally.

Human rhinovirus (HRV) infections are typically associated with mild upper respiratory tract symptoms. However, in some cases, especially in children, it can lead to more severe respiratory diseases, particularly in patients with chronic pulmonary disease, and immunocompromised hosts^4^.

Dengue is an arboviral disease caused by a virus (family *Flaviviridae*, genus *Flavivirus*) with four serotypes (DENV-1 to DENV-4), being transmitted primarily by *Aedes* mosquitoes^5^. Dengue fever is generally self-limiting and rarely fatal (case fatality = 0.03%, according to the Brazilian Ministry of Health)^5^. This disease has reemerged in recent years, with an expanded geographic distribution of both the virus and its mosquito vectors, increased epidemic activity, and the development of hyperendemicity (cocirculation of multiple serotypes)^5^.

Leptospirosis, dengue, and COVID-19 all show a wide range of clinical manifestations, from mild, self-limited febrile illnesses to severe conditions affecting multiple systems^1–3,5^.

It remains unclear whether coinfection with HRVs and other respiratory viruses could aggravate associated symptoms^6^.

There is little information in the medical literature regarding such coinfections and their potential impact on clinical progression and outcomes^7^. These diseases often have nonspecific initial symptoms, which can lead to delayed diagnosis and treatment^7^.

Here, we report a well-documented coinfection case involving three infectious agents, *Leptospira* spp., SARS-CoV-2, and an HRV, as well as a recent infection (acute phase) with dengue virus (DENV) in a previously healthy woman with no history of chronic or malignant diseases. She initially presented with acute febrile jaundice, which rapidly progressed to pulmonary hemorrhage and multiple organ system dysfunction syndrome, ultimately leading to a fatal outcome.

### Ethics

All data were generated as part of routine work at the Hospital Municipal Antonio Giglio, Osasco, Sao Paulo State, Brazil. The study was approved by institutional ethical committee (Plataforma Brasil, N° 7.060.293).

## CASE REPORT

A previously healthy 33-year-old woman, with no history of chronic or malignant diseases, was admitted to the emergency room (ER) of the Municipal Public Hospital (Osasco, Sao Paulo State, Brazil; metropolitan region of Sao Paulo city) with complaints of a four-day fever and one day of jaundice and dyspnea. She had received an incomplete COVID-19 vaccination regimen (three doses; last dose in November 2022) and had not been vaccinated against dengue. She also reported contact with floodwater a few days prior to the onset of symptoms.

Other positive findings on clinical examination included tachycardia, hypotension, dehydration, cyanosis, crackles in both lungs, abdominal pain, myalgia, oliguria, rash, petechiae, and edema in both legs. Laboratory tests revealed thrombocytopenia, lymphopenia, hypoxemia, acute kidney injury, rhabdomyolysis, hyperbilirubinemia, and an elevated C-reactive protein concentration (Table 1).

**Table 1:** Patient laboratory data obtained upon admission to the emergency room and intensive care unit.

Variable	Reference Range	Admission (ER)	Admission (ICU)
Hematocrit (%)	35–50	39.8	35.8
Hemoglobin (g/dL)	11.0–16.5	15.8	12.4
Erythrocytes (x10^6^ per mm^3^)	4.2-5.4	4,19	-
Leukocytes (count per mm³)	5.0–10	24,000	10,500
C-reactive Protein (mg/dL)	<1.0 mg/dL	14.9	88.4
**Differential**			
Granulocytes	2500–7000	-	8,899
Lymphocytes	1000–4000	-	431
Monocytes (%)	4.0–10	-	3.6
Platelets (count per mm³)	150,000–4,500,000	41,000	21,000
Erythrocytes (count per mm³)	3,800,000–5,800,000	-	-
Total Bilirubin (mg/dL)	0–1.1	-	8.9
Indirect Bilirubin (mg/dL)	0–0.75	-	1.9
Direct Bilirubin (mg/dL)	0–0.25	-	7.0
Urea (mg/dL)	4.7–23	94	114.0
Creatinine (mg/dL)	0.9–1.3	3.5	3.5
Creatine Kinase (U/L)	26–140	-	2,135
Serum Glucose (mg/dL)	70–99	145	47
**Arterial Blood Gases**			
Inspired Oxygen Fraction	0.21%	-	1.0%
pH	7.3–7.4	-	7.39
PaCO_2_ (mmHg)	38–50	-	21.5
Bicarbonate (mEq/L)	22–26	-	4.4
PaO_2_ (mmHg)	35–50	-	137

- = not available;

ER = emergency room; ICU = intensive care unit. FiO_2_ =100% (pO2/FiO_2_ ratio = 137).

**Figure 1:**
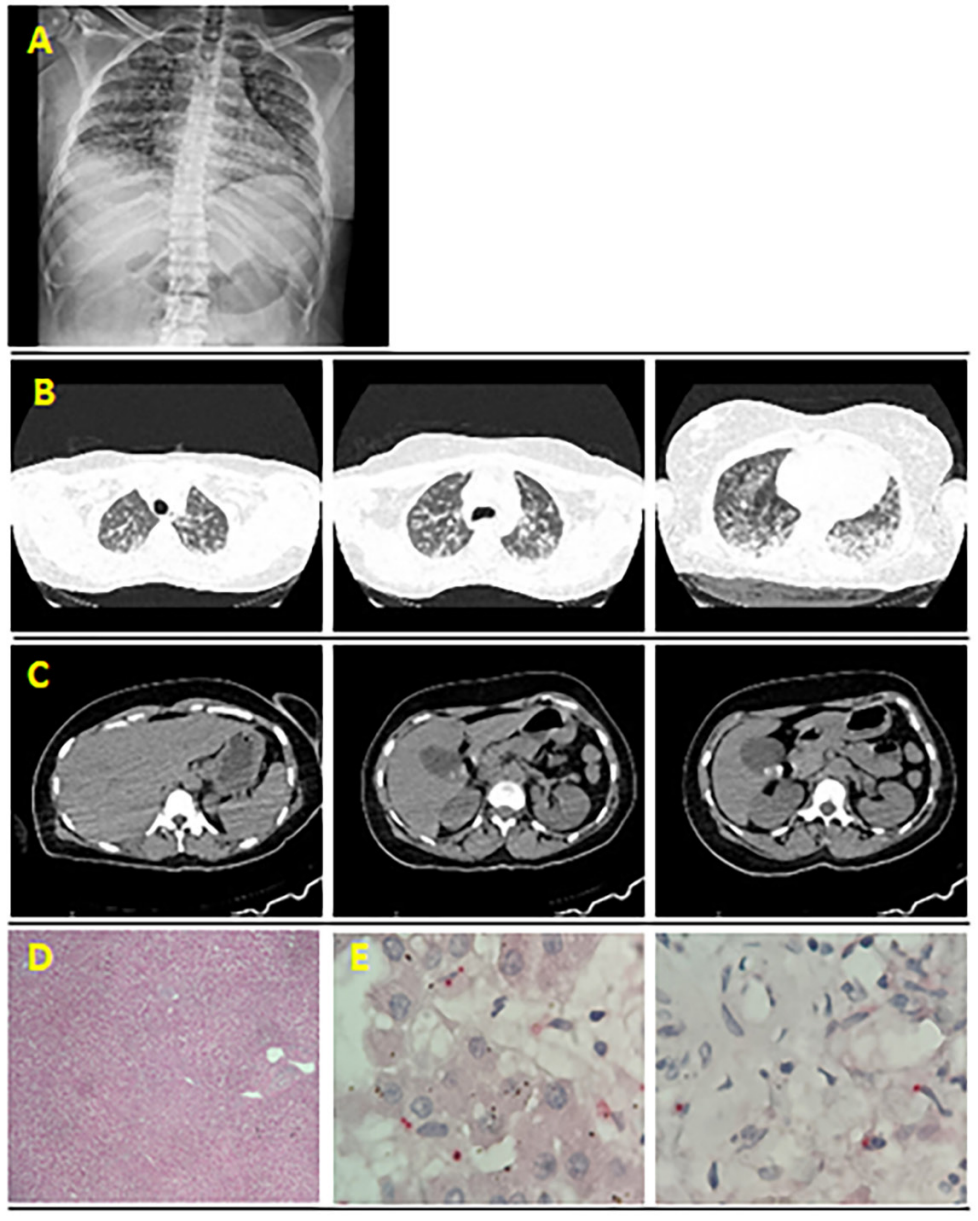
(A) Chest X-ray reveals bilateral opacities suggestive of an inflammatory process; (B) High-resolution chest CT scan reveals bilateral pulmonary changes with an acute inflammatory appearance affecting more than 50% of the parenchyma. No pleural effusion is observed. No evidence of mediastinal lymphadenopathy can be observed. The mediastinal vascular structures are of normal caliber and course; (C) Abdominal CT scan reveals dense stones measuring up to 1.2 cm within the gallbladder, parietal thickening that may be related to an inflammatory/infectious process, and no biliary tract dilatation; (D) Histological image of the liver at 40x and 100x magnification (H&E stain) reveals several significant findings. Mild disarray is observed in the liver cell plates, indicating slight architectural disruption among the hepatocytes. The sinusoids are congested and dilated and exhibit increased blood content. In the portal areas, a mild mixed inflammatory reaction is present, characterized by an infiltrate consisting of lymphocytes, plasma cells, and occasional neutrophils. These histological features collectively reflect the liver's response to leptospiral infection, highlighting structural changes and inflammatory activity; (E) Immunohistochemical analysis reveals a distinct, pink granular and cytoplasmic pattern of positivity for *Leptospira* in Kupffer cells and the portal track (phosphatase alkaline, 1000x).

Chest radiography (Figure 1A) revealed bilateral lung involvement and signs of alveolar edema, with lesions mainly confined to the middle and lower zones of both lungs. High-resolution tomography of the chest revealed bilateral ground-glass opacities (Figure 1B). Computed tomography of the abdomen revealed cholelithiasis with gallbladder wall thickening, suggestive of an inflammatory process (Figure 1C).

She was treated with oxygen therapy, antibiotics, and intravenous saline solution for volume expansion.

The patient's respiratory symptoms worsened as she developed hemoptysis. Invasive mechanical ventilation was initiated immediately after admission to the intensive care unit (ICU). She died four hours after hospital admission.

Nasopharyngeal swab samples and blood were collected and analyzed to test for the presence of a range of pathogens (Table 2).

**Table 2:** Targeted laboratory findings for infectious agents.

Agent	Methodology	Clinical Sample	Result
Zika Virus**	RT‒qPCR	Blood	Not detectable
Chikungunya**	RT‒qPCR	Blood	Not detectable
Dengue**	RT‒qPCR	Blood	Not detectable
Dengue (IgM)	MAC-ELISA	Blood	**Reagent**
Adenovirus*	RT‒qPCR	Nasopharyngeal swab	Not detectable
Influenza A*	RT‒qPCR	Nasopharyngeal swab	Not detectable
Influenza A***	RT‒qPCR	Liver (FFPE)	Not detectable
Influenza B*	RT‒qPCR	Nasopharyngeal swab	Not detectable
Influenza B***	RT‒qPCR	Liver (FFPE)	Not detectable
Human Rhinovirus*	RT‒qPCR	Nasopharyngeal swab	**Detectable**
RSV*	RT‒qPCR	Nasopharyngeal swab	Not detectable
RSV***	RT‒qPCR	Liver (FFPE)	Not detectable
SARS-CoV-2*	RT‒qPCR	Nasopharyngeal swab	**Detectable**
SARS-CoV-2***	RT‒qPCR	Liver (FFPE)	**Detectable**
Leptospira	MAC-ELISA	Blood	**Reagent**
Leptospira	qPCR	Blood	**Detectable**
Leptospira	qPCR	Liver (FFPE)	**Detectable**
Leptospira	Immunohistochemistry	Liver (FFPE)	**Positive**
Bacteria	Blood Culture	Blood	Negative

RT = reverse transcriptase reaction; qPCR = real-time polymerase chain reaction; FFPE = formalin-fixed and paraffin-embedded; IgM = immunoglobulin M; MAC-ELISA: IgM = antibody capture enzyme-linked immunosorbent assay; RSV = respiratory syncytial virus;

* Kit: CDC (Atlanta/USA);

** Kit: TaqMan Zika Virus Triplex Kit (Thermo Fisher Scientific, Massachusetts, USA);

*** Kit: Allplex™ SARS-CoV-2/FluA/FluB/RSV Assay (Seegene, Seoul, KR).

The nasopharyngeal swabs were tested via reverse transcriptase polymerase chain reaction (RT–PCR) for COVID-19 and other respiratory viruses (influenza virus, respiratory syncytial virus [RSV] and HRVs). Results for influenza A and B and RSV were negative, but SARS-CoV-2 and HRV were detected (Table 2).

Upon hospital admission, blood cultures and immunological tests for arboviruses (dengue, Zika, and chikungunya) and leptospirosis were also conducted. Blood cultures revealed no bacterial growth, but the IgM antibody capture enzyme-linked immunosorbent assay (MAC-ELISA) was positive for DENV and *Leptospira* spp. Immunological tests for chikungunya and Zika viruses were negative.

In the postmortem analysis, a liver tissue sample revealed disarrayed hepatocytes, lymphomonocytic inflammatory infiltrate, and the presence of both *Leptospira* spp. and SARS-CoV-2, which were confirmed after immunohistochemical staining (Figures 1D and 1E).

All laboratory investigations were performed at the Adolfo Lutz Institute, Sao Paulo State, Brazil, the country's reference laboratory.

## DISCUSSION

Reports of coinfection with leptospirosis and other infectious agents pose a challenge in clinical practice^7^, likely due to difficulties in isolating these agents, symptom overlap, and lack of specific investigation protocols in regions with emerging and re-emerging diseases^7^.

Leptospirosis and COVID-19 are common diseases in Osasco city (part of the Sao Paulo metropolitan area), while Brazil is currently facing a major dengue epidemic with a high mortality rate.

The patient had received an incomplete COVID-19 vaccination regimen and was not vaccinated against dengue. She had been exposed to contaminated water days before the onset of symptoms, which is considered a risk factor for leptospirosis.

Upon presentation, the patient showed liver involvement (jaundice), rhabdomyolysis, oliguric renal failure, respiratory symptoms (including severe hypoxemia), and hemorrhagic manifestations (hemoptysis, petechiae, skin rash, and thrombocytopenia). These findings are characteristic of severe leptospirosis (Weil's disease), severe forms of dengue, and severe COVID-19^1–3,5,8–14^.

The detection of SARS-CoV-2 and *Leptospira* ssp. in liver tissue via RT–qPCR suggested the systemic dissemination of the viruses and bacterium.

Radiological findings, which showed bilateral alveolar infiltrates without pleural effusion, are common in both leptospirosis and COVID-19 patients (Figures 1A and 1B)^1–3,10^. Coinfection with leptospirosis and COVID-19 has also been associated with poor outcomes in coinfected patients compared to those with either infection alone^15^.

Although RT–PCR results for dengue were negative, the presence of a rash, petechiae, hemoconcentration, thrombocytopenia, gallbladder inflammation (Figure 1C), and IgM antibodies in the blood (MAC-ELISA) suggested a recent DENV infection^5,8,13,14^. Severe respiratory involvement is uncommon in dengue; however, chest CT findings, including pleural effusion and signs of pulmonary edema, were probably associated with increased vascular permeability^16^.

HRVs typically cause mild upper respiratory tract infections, and coinfection with other respiratory viruses has been documented^4^. Coinfection with SARS-CoV-2 may exacerbate the severity of the infection^6^.

Although these infectious agents have different pathophysiological mechanisms and target different organs, their co-occurrence may have accelerated disease progression via cytokine release and endothelial injury. This could have led to vasculopathy, coagulopathy, and capillary leakage, ultimately causing multiorgan and system dysfunction^1–3,5^.

Despite initial treatment with antibiotics, fluid replacement, ventilatory support, and intensive care, the patient experienced a rapid and fatal outcome.

One strength of this case report is that it highlights the potential for coinfections to occur and contribute to fatal outcomes, especially in regions with emerging and re-emerging diseases. Our manuscript underscores the importance of diagnostic tools in detecting atypical conditions in patients with acute febrile syndrome in endemic areas, aiming to enhance routine diagnoses and minimize post-mortem ones.

Another strength is that all tests were conducted in a reference laboratory for emerging and re-emerging diseases, while a thorough investigation was performed at the admitting hospital.

One limitation of this report is that the material submitted for histopathological analysis was restricted to a liver tissue sample. No other tissues from other target organs (e.g., lung, kidney, or lymph nodes) were sent for analysis.

Adopting prophylactic measures (such as avoiding contaminated soil, wearing protective boots, and completing vaccination and booster schedules) and establishing investigation protocols for febrile jaundice syndrome patients in endemic areas could facilitate early diagnosis and administration of specific treatment^1–3,5,10,12^.

## CONCLUSION

Coinfection with leptospirosis and other diseases are rarely described in the literature, likely due to challenges in isolating responsible agents, symptom overlap, and lack of specific investigation protocols in regions with emerging and re-emerging diseases. Coinfection with several infectious agents can exacerbate the progression of individual diseases. Research protocols for such diseases could aid in early diagnosis and treatment. Prophylactic measures, including vaccination campaigns and avoiding contact with contaminated water and soil, could reduce the impact of these potentially fatal diseases.
